# Dr. James L. Stewart

**Published:** 1891-01

**Authors:** 


Dr. James L. Stewart, of Erie, Pa., died in that city December 6, 1890, aged sixty-six years. Dr Stewart served as a Surgeon of Pénnsylvania Volunteers during the war, and afterward achieved considerable local renown as a surgeon. He had but lately returned from the Berlin Medical Congress, when his fatal illness seized him. He had held office in the several local, State and National Medical Societies, and was a conspicuous figure in the medical and social circles of his city.

